# Lipopolysaccharide Associates with Amyloid Plaques, Neurons and Oligodendrocytes in Alzheimer’s Disease Brain: A Review

**DOI:** 10.3389/fnagi.2018.00042

**Published:** 2018-02-22

**Authors:** Xinhua Zhan, Boryana Stamova, Frank R. Sharp

**Affiliations:** Department of Neurology, MIND Institute, University of California, Davis, Davis, CA, United States

**Keywords:** Alzheimer’s disease, lipopolysaccharide, cytokines, TLR4, myelin, MBP, oligodendrocytes, amyloid plaque

## Abstract

This review proposes that lipopolysaccharide (LPS, found in the wall of all Gram-negative bacteria) could play a role in causing sporadic Alzheimer’s disease (AD). This is based in part upon recent studies showing that: Gram-negative *E. coli* bacteria can form extracellular amyloid; bacterial-encoded 16S rRNA is present in all human brains with over 70% being Gram-negative bacteria; ultrastructural analyses have shown microbes in erythrocytes of AD patients; blood LPS levels in AD patients are 3-fold the levels in control; LPS combined with focal cerebral ischemia and hypoxia produced amyloid-like plaques and myelin injury in adult rat cortex. Moreover, Gram-negative bacterial LPS was found in aging control and AD brains, though LPS levels were much higher in AD brains. In addition, LPS co-localized with amyloid plaques, peri-vascular amyloid, neurons, and oligodendrocytes in AD brains. Based upon the postulate LPS caused oligodendrocyte injury, degraded Myelin Basic Protein (dMBP) levels were found to be much higher in AD compared to control brains. Immunofluorescence showed that the dMBP co-localized with β amyloid (Aβ) and LPS in amyloid plaques in AD brain, and dMBP and other myelin molecules were found in the walls of vesicles in periventricular White Matter (WM). These data led to the hypothesis that LPS acts on leukocyte and microglial TLR4-CD14/TLR2 receptors to produce NFkB mediated increases of cytokines which increase Aβ levels, damage oligodendrocytes and produce myelin injury found in AD brain. Since Aβ_1–42_ is also an agonist for TLR4 receptors, this could produce a vicious cycle that accounts for the relentless progression of AD. Thus, LPS, the TLR4 receptor complex, and Gram-negative bacteria might be treatment or prevention targets for sporadic AD.

## Introduction

Rare early-onset familial forms of Alzheimer’s disease (AD) are associated with autosomal dominant mutations in the amyloid beta precursor protein (AβPP), presenilin 1 and presenilin 2 genes (Goate and Hardy, 2012). Mouse models based upon these mutated human genes have guided much of the drug discovery for AD (Belkacemi and Ramassamy, 2012; Hall and Roberson, 2012). However, clinical trials based upon these models have yet to lead to successful treatments (Selkoe, 2011; Huang and Mucke, 2012; Cavanaugh et al., 2014). This has raised questions about the “amyloid hypothesis” for sporadic AD (Herrup, 2015).

An enigma in the AD field has been the inability to identify a cause(s) for the much more common sporadic late onset alzheimer’s disease (LOAD/AD). This is important because neither β amyloid (Aβ) nor abnormal tau may “cause” sporadic AD, but rather could be downstream of an unrelated primary pathological process. In the first part of this brief review some epidemiological and neuropathological findings that do not appear to have a direct connection with the amyloid hypothesis are summarized. In the second portion of the review, data are summarized showing Lipopolysaccharide (LPS) in human brain and greater amounts of LPS in AD brain that are associated with amyloid plaques, perivascular amyloid and neurons. In the third portion, recent studies showing the presence of myelin injury in all AD compared to control brains are reviewed, and the association of LPS with oligodendrocytes which could injure oligodendrocytes and myelin. In the final fourth portion of the review a simple model is presented by which LPS acts on TLR4/CD14 receptors to activate NFkB and increase cytokines which contribute to increasing Aβ in AD brain and producing myelin injury including formation of degraded Myelin Basic Protein (dMBP). Since LPS and Aβ are both agonists for the TLR4/CD14 receptor (Lehnardt et al., 2002; Vollmar et al., 2010; Scott et al., 2017), this could set up a vicious cycle where LPS acts on the TLR4/CD14 receptor which increases Aβ which in turn provides positive feedback on the TLR4/CD14 receptor to produce progressive injury in AD brain. TLR4-TLR2 downstream interactions are not discussed to limit the scope of the review.

## Epidemiological and Biological Factors in AD

### Inflammation

Inflammation has repeatedly been implicated in AD but the source for this inflammation has been elusive. Inflammatory proteins in blood, notably C reactive protein (CRP) and IL6, are elevated several years before the clinical onset of dementia in several studies (Schmidt et al., 2002; Engelhart et al., 2004; Tilvis et al., 2004; Kuo et al., 2005). A high plasma CRP has been associated with a 3-fold increased risk of developing AD years later (Schmidt et al., 2002). Another study showed cognitively intact older individuals in the top tertile for leukocyte IL1β or TNFα production have ~3 times increased risk of developing AD compared to those in the lowest tertile (Tan et al., 2007). Though clinical trials have shown non-steroidal anti-inflammatory drugs (NSAIDs) do not affect cognitive decline in AD (de Craen et al., 2005; Imbimbo, 2009; Imbimbo et al., 2010), meta-analyses show regular NSAID use is associated with a 2 fold reduction in the odds of developing AD (McGeer et al., 1996). Moreover, a large case control study (>200,000 subjects) showed that the regular use of NSAIDs reduced the risk of developing AD (Vlad et al., 2008). Thus, NSAIDS may delay the onset of AD, but once it develops NSAIDS do not appear to affect the course of AD, suggesting an opportunity for intervention prior to disease onset (Grammas, 2011; Butchart and Holmes, 2012). These data suggest that inflammation is occurring for some time prior to onset of AD pathology and symptoms, but there has been little indication of what drives the inflammation that goes on for years. This review proposes this may be due to Gram-negative bacterial LPS in blood and in brain of AD subjects.

### Infection

Delirium, which is often caused by infection, is associated with increased incidence of subsequent development of dementia in cognitively intact old individuals (Rahkonen et al., 2000a,b). The presence of one or more infections over a 5-year follow up period increased the odds of developing AD, and risk increased with age (Dunn et al., 2005). Receiving DPT vaccines early in life as well as other vaccines later in life significantly reduces the risk of subsequent AD (Tyas et al., 2001; Verreault et al., 2001). Protection by DPT vaccine may be due in part to preventing infection by the Gram-negative Bordetella Pertussis bacterium which causes whooping cough. Tooth loss (Stein et al., 2007) and oral infections (Poole et al., 2013; Abbayya et al., 2015; Chen et al., 2017) have been associated with AD. Furthermore, *Porphyromonas gingivalis* (Ishida et al., 2017) and LPS from *Porphyromonas gingivalis* (Wu et al., 2017) produce AD-like phenotypes in mice. In addition, Spirochetes (Miklossy, 1993; Miklossy et al., 1994, 2004; Riviere et al., 2002), chlamydophila pneumonia (Hammond et al., 2010), Helicobacter pylori (Kountouras et al., 2009), fungi (Pisa et al., 2015), herpes viruses (Civitelli et al., 2015) and cytomegalovirus (Lovheim et al., 2018) are also reported to be involved in with AD pathology. Interestingly, previous *in vitro* studies demonstrate that amyloid-like morphological changes occur following Borrelia burgdorferi spirochetes or LPS exposure. These studies suggest that AD might be a neurological disorder associated with infectious agents.

It is possible that several different infections might initiate downstream AD pathology. The possibility of infection received a significant boost with the recent discovery that every human brain examined had evidence of Gram-negative bacteria (Branton et al., 2013; Emery et al., 2017) though the issue of contamination in these studies has yet to be resolved. For this review the focus is on Gram-negative bacterial LPS as it is associated with and may cause AD pathology.

### Neurovascular Abnormalities in AD

There is considerable evidence for vascular abnormalities in AD. Cerebral blood flow is reduced in AD prior to cognitive decline (Ruitenberg et al., 2005). Patients with an APOE4 allele have disrupted fMRI connectivity (blood flow correlations) in the absence of amyloid plaques (detected by PET) or decreased CSF Aβ_42_ (Sheline et al., 2010). Cerebral glucose metabolism is decreased in preclinical and prodromal AD before symptoms (Hunt et al., 2007; Herholz, 2010). Microvessels isolated from AD patients release many cytokines, chemokines and proteases compared to control patients (Grammas, 2011). In genetic mouse models of AD, alterations of blood flow and BBB permeability occur before symptoms and before amyloid beta deposition (Iadecola et al., 1999; Ujiie et al., 2003; Iadecola, 2004). Using vascular corrosion casts, the 3D arrangement of the brain vessels is abnormal in a mouse model of AD prior to appearance of amyloid plaques (Meyer et al., 2008). In addition, BBB breakdown has been demonstrated in humans with mild cognitive impairment (MCI) and early AD before brain atrophy and dementia (Montagne et al., 2017). These data provide strong evidence for neurovascular abnormalities in sporadic AD in humans and in mouse genetic AD models prior to appearance of brain amyloid plaque neuropathology (Zlokovic, 2005, 2008, 2011) and support the fact that both cardiovascular disease and cerebrovascular disease are significant risk factors for sporadic AD (Tyas et al., 2001; Veurink et al., 2003; White et al., 2005; Zhu et al., 2007; Marlatt et al., 2008; de la Torre, 2009; Guglielmotto et al., 2009). Notably, cerebral vascular disease and AD pathology co-exist in up to 80% of aging human brains (Schneider et al., 2007; Savva et al., 2009; Iadecola, 2013). These data are relevant for our finding of LPS in brain described below, since areas of ischemic and/or hypoxic injury might provide a portal for LPS entry from blood into human brain.

Recent neuroimaging studies in individuals with MCI and early AD have shown BBB breakdown in the hippocampus (Montagne et al., 2015) and several Gray Matter (GM) and White Matter (WM) regions (van de Haar et al., 2016a,b, 2017).

### Genetic Variants

Besides the original discovery of the association of Apolipoprotein E4 with sporadic AD (Bertram et al., 2010; Bertram and Tanzi, 2012; Goate and Hardy, 2012), additional genetic insights into sporadic AD have been made using GWAS (Bertram et al., 2010; Bertram and Tanzi, 2012). The genes implicated in these studies are involved in Aβ clearance at the blood brain barrier (APOE, BIN1, CLU, CR1, PICALM; Zlokovic et al., 1996; O’Brien and Wong, 2011; Wu et al., 2012), in inflammation in blood and brain (APOE, CD33, CLU, CR1, HLA-DRB5/1, INPP5D, TREM2; Bertram et al., 2010; Veerhuis, 2011; Bertram and Tanzi, 2012; Crehan et al., 2012), in the immune response (CR1, CD33, MS4A, CLU, ABCA7, EPHA1, HLA-DRB5-HLA-DRB1), endocytosis (BIN1, PICALM, CD2AP, EPHA1 and SORL1) and lipid biology (CLU, ABCA7 and SORL1; Karch and Goate, 2015). Many of these genes are expressed by peripheral monocytes and brain microglia (Villegas-Llerena et al., 2016) and could be associated with an infectious cause of sporadic AD (Heneka et al., 2015). Our model of how LPS could lead to AD neuropathology could incorporate all these genes and molecules (see below). Since Aβ also acts on TLR4 receptors on both monocytes and neutrophils, this could help explain how neutrophils promote AD-like pathology with cognitive decline via the leukocyte LFA-1 adhesion molecule in two AD mouse models (Zenaro et al., 2015).

### Myelin Injury in AD Brain

Myelin injury has been recognized in AD brain for some time, including the very first report of AD pathology by Alzheimer (Alzheimer et al., 1991; Scheltens et al., 1995; Englund, 1998; Möller and Graeber, 1998; Bartzokis, 2011). The relationship of the myelin injury to the other better-known neuropathology of AD, including amyloid plaques and tau-neurofibrillary tangles, has been unclear and the explanation for such myelin injury has been equally obscure. However, recently myelin injury has been suggested as an important/principal component of AD pathophysiology. The volume of White Matter Hyperintensities (WMH) in AD brain predicts the rate and severity of cognitive decline (Brickman et al., 2008). The Low-density lipoprotein receptor-related protein 1, which transports Aβ out of brain, is also an essential receptor for myelin phagocytosis providing a link between myelin damage and Aβ (Gaultier et al., 2009). Moreover, Aβ_1–42_ inhibits myelin sheet formation *in vitro* (Horiuchi et al., 2012). Aβ_1–42_ directly binds myelin basic protein (MBP; Liao et al., 2009, 2010; Kotarba et al., 2013). There is a focal loss of oligodendrocytes and myelin within and adjacent to amyloid plaques in familial and sporadic AD brain (Mitew et al., 2010). However, the absence of MBP decreases the accumulation of Aβ_1–42_ in transgenic AD mice, and essentially eliminate amyloid plaques (Ou-Yang and Van Nostrand, 2013). More recently it has been discovered that loss of ceramide synthase 2 activity, necessary for myelin biosynthesis, precedes tau and amyloid pathology in human AD cortex (Couttas et al., 2016). In addition, oligodendrocyte and myelin injury can precede the formation of amyloid plaques and tau pathology in a mouse AD model (Mitew et al., 2010; Hall and Roberson, 2012). Clinically, WMH are more highly associated with preclinical AD than imaging and cognitive markers of neurodegeneration; and WMH are now considered a core feature of dominantly inherited AD (Desai et al., 2010; Kandel et al., 2016; Lee et al., 2016). Of interest, there are very high titers of autoantibodies against a variety of myelin proteins in blood of AD patients compared to controls (Papuc et al., 2015). The cause of the myelin injury in AD brain, however, remains to be elucidated. This review hypothesizes that Gram negative bacterial LPS molecules are present in AD WM and GM where they bind oligodendrocytes and cause an increase in cytokines and oxidative stress that contributes directly to damage of oligodendrocytes and to myelin proteins including MBP which then associate with Aβ_1–42_ in amyloid plaques in AD brain.

### Microbiome of the Gut and AD

There is emerging evidence that the gut microbiome affects neurological diseases including AD. There is a different gut microbiome in wild type mice compared to mouse AD models (Shen et al., 2017) and in control compared to AD patients (Vogt et al., 2017). There is an association of brain amyloidosis with pro-inflammatory gut bacterial taxa and peripheral inflammation markers in cognitively impaired elderly humans (Cattaneo et al., 2017). As people age they have more Gram-negative bacteria in the gut. Antibiotic-induced perturbations in gut microbial diversity influences neuro-inflammation and amyloidosis in a murine model of AD (Minter et al., 2016). Serum IgG antibody levels to periodontal microbiota, some derived from the gut, are associated with incident AD (Noble et al., 2014). The gut microbiome is implicated in normal neurodevelopment, autism spectrum disorders, schizophrenia, depression, Parkinson’s, multiple sclerosis, stroke and aging (Branton et al., 2016; Sampson et al., 2016; Sharon et al., 2016; Winek et al., 2016). Though there is a marked increase in reports on the gut microbiome affecting neuropsychiatric diseases, very few have developed a cogent hypothesis on how this occurs. This proposal hypothesizes that Gram-negative bacteria, potentially from the gut or gums as well as from systemic infections, all release LPS which is engulfed via TLR4-CD14 receptors by blood leukocytes including monocytes and neutrophils, and by brain microglia. The TLR4 mediated activation of NFkB increases cytokines which contribute to myelin damage. Studies have demonstrated that NFkB pathway regulates the activity of Beta-secretase 1 (BACE1; Buggia-Prevot et al., 2008; Guglielmotto et al., 2012), the crucial enzyme for Aβ production and the BACE1 level in AD brain is increased compared to control (Guglielmotto et al., 2012). These studies suggest that activation of NFkB may contribute to increases of Aβ and amyloid pathology in AD brain.

## LPS in Human AD Brain

Based upon the above considerations, and based upon genomic studies of blood of AD patients showing evidence of inflammation, oxidative stress and hypoxia (Bai et al., 2014), the hypothesis was developed that several systemic factors act together to produce AD (Zhan et al., 2015a). There must be some cause of inflammation, it was reasoned that either an infectious agent or molecules from infectious agents might be important in causing AD, in particular lipopolysaccharide (LPS) which is found in the outer wall of all Gram-negative bacteria. This was done for several reasons. (1) LPS containing *E. coli* bacteria can form extracellular amyloid (Blanco et al., 2012; Hill and Lukiw, 2015). (2) A recent study showed bacterially-encoded 16S rRNA sequences in all human brain specimens with Gram-negative, LPS containing alpha-proteobacteria representing over 70% of the bacterial sequences (Branton et al., 2013). (3) Ultrastructural analysis by scanning electron microscopy confirmed the presence of microbes in erythrocytes of AD patients (Bester et al., 2015; Potgieter et al., 2015). (4) Another study demonstrated that blood LPS levels in AD patients are 3-fold the levels in control (Zhang et al., 2009). (5) In addition, we developed a rat model where LPS was combined with focal cerebral ischemia and hypoxia (LPS-IS-HY; Zhan et al., 2015a). This combination was chosen because it causes myelin injury in newborn rodent brain (Hagberg et al., 2002; Lehnardt et al., 2002, 2003; Pang et al., 2003) and evidence of WM injury is found in every human AD brain (Zhan et al., 2014, 2015b). The combination of LPS, focal ischemia and hypoxia in adult rats produced: (a) increases of cytokines in brain; (b) myelin injury in the ischemic and non-ischemic hemispheres; and (c) formation of amyloid-like plaques where degraded MBP, AβPP and Aβ co-localized. These data led to the search for LPS and Gram-negative bacteria in human AD brain.

Gram-negative bacteria are one of the most important causes of human infectious diseases including gastroenteritis (*Escherichia coli, Shigella, Salmonella, Vibrio cholera*), pulmonary infections (*Klebsiella pneumoniae, Legionella, Pertussis*/whooping cough, *Pseudomonas aeruginosa*), urinary tract infections (*Escherichia coli, Proteus mirabilis, Enterobacter cloacae, Serratia marcescens, Bacteroides*), ulcers (*Helicobacter pylori*), sexually transmitted disease (*Neisseria gonorrhoeae*), meningitis (*Neisseria meningitidis*) and gum/periodontal disease (*Porphyromonas gingivalis*). Gram-negative bacteria are also resident in the normal gut and increase in numbers with age (Sharon et al., 2016). Gram-negative bacterial periodontal disease has been repeatedly associated with AD (Noble et al., 2014; Kamer et al., 2015; Olsen and Singhrao, 2015). The evidence that blood LPS levels in AD patients are 3-fold the levels in control (Zhang et al., 2009) also suggests that LPS associates with AD.

**Figure 1:**
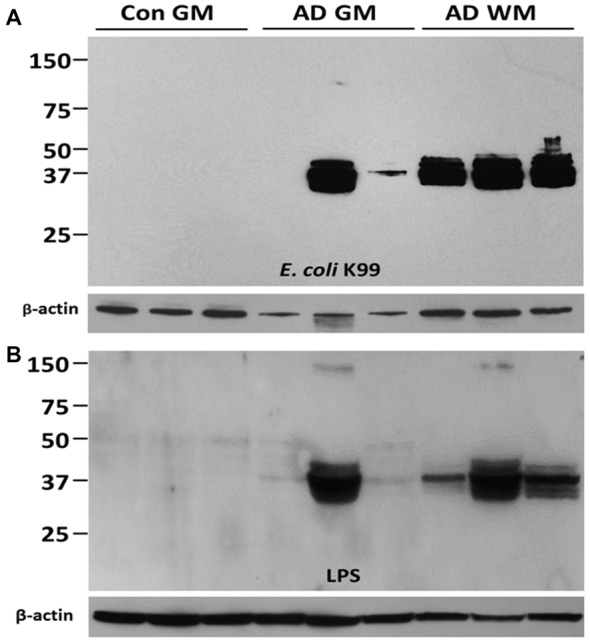
Western Blot analysis of Gram-negative bacterial molecules in human brain. **(A)** Western blots for *E. coli* K99 pili protein showed K99 protein in 3/3 Alzheimer’s disease (AD) White Matter (WM) samples, 2/3 AD Gray Matter (GM) samples and 0/3 Control GM samples. β-actin was used as loading control. **(B)** Western blots for Gram-negative bacteria lipopolysaccharide (LPS) in 3/3 AD WM samples, 3/3 AD GM samples and 0/3 Control GM samples. β-actin was used as loading control. This Figure is from Zhan et al. (2016). Reproduced with permission.

**Figure 2:**
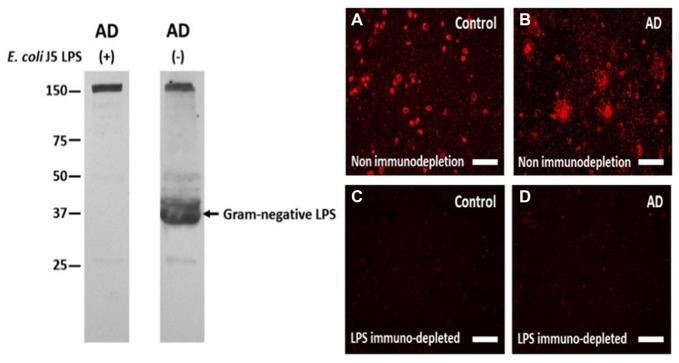
The antibody to LPS stains LPS in human brain on Western blots and using immunofluorescence, and immunofluorescence can be eliminated by immunoprecipitating the antibody with excess LPS. Left Panel: the antibody to LPS stains one large band on a Western blot (AD (−) lane) which is eliminated when the antibody was immunoprecipitated with excess *E. coli* J5 LPS (AD (+) lane). The band at 150 kD in both lanes shows equal protein loading in the two lanes. Right Panel: The antibody to LPS produced more immunofluorescence in AD cortex **(B)** compared to control cortex **(A)**. After immunodepletion, the immunofluorescence in AD **(D)** and Control **(C)** cortex were eliminated. These are supplementary figures from Zhan et al. (2016). Reproduced with permission.

Based upon the above literature and the rat model findings, a search for LPS and other *E. coli* molecules in human AD and control brains was undertaken. Studying LPS is problematic since it is so pervasive in the environment, and is a common contaminant of solutions in the laboratory. Thus, the studies were performed with endotoxin free reagents, and multiple controls performed to ensure no LPS contamination of the reagents used. Western blots were performed for LPS and the K99 pili protein derived from *E. coli* (Zhan et al., 2016). Figure 1A shows the presence of *E. coli* K99 pili protein in two of three AD superior temporal GM samples, and in three of three AD frontal lobe WM samples and none detectable in three control GM samples. Figure 1B shows LPS in the same three AD GM and three AD WM samples compared to none detectable by Western blot in the three control GM samples (Zhan et al., 2016). In Figure 2 the Western blots (on the left) show LPS stained from AD brain (−) and elimination of the LPS band using immunodepletion with excess LPS (AD (+)). The stained band at ~150 kD shows equal protein loading in the two lanes.

To localize LPS at the cellular level immunolabeling was performed using the same antibody as used for the Western blots. There was LPS immunofluorescence in control (Figure 2A) and in AD brain (Figure 2B), but with LPS^+^ aggregates only observed in AD brain (Figures 2A,B). Immunofluorescence with the antibody to LPS, but immunodepleted with LPS, showed no fluorescent staining in control (Figure 2C) or AD brain (Figure 2D). These findings were important for showing LPS aggregates in AD brains. The lack of staining on Western blots for LPS in control brains (Figure 1B, left panels) is likely accounted for by the lower sensitivity of Western blots compared to immunofluorescence.

**Figure 3:**
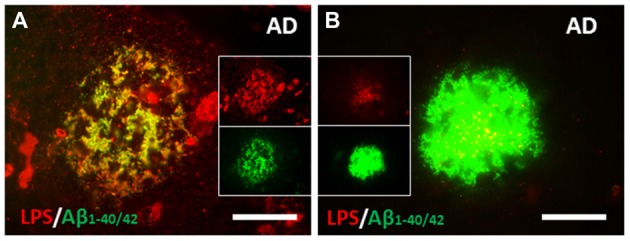
Co-localization of LPS and Aβ in human AD brain. **(A)** There were large clusters of LPS that co-localized with Aβ_1–40/42_ in some amyloid plaques. **(B)** The most common pattern, however were confluent Aβ_1–40/42_ stained plaques that had LPS stained particles (yellow on merged image) within them. **(A,B)** are from Zhan et al. (2016). Reproduced with permission.

The relationship of LPS to amyloid plaques was examined next. LPS co-localized with Aβ in AD brain (Figure 3) and LPS co-localized with perivascular amyloid in AD brain (not shown). There were several different patterns of co-localization of LPS and Aβ_1–40/42_ in AD brains from more LPS compared to Aβ_1–40/42_ (Figure 3A) to more Aβ_1–40/42_ compared to LPS (Figure 3B) in the amyloid plaques. Not shown in the figures here was the finding of LPS in the nucleus of neurons in AD brains (Zhan et al., 2016).

In addition to superior temporal GM and frontal lobe WM of AD brains, the same LPS antigen is found in the hippocampus of AD brains as well (Zhao et al., 2017a,b). Moreover, one previous study demonstrated that LPS from *Porphyromonas gingivalis* is present in some AD brains as well (Poole et al., 2013). Thus, it is possible that LPS from multiple strains of Gram negative bacteria might be involved in AD pathology.

## Degraded MBP (dMBP) in AD Compared to Control Brains

### dMBP Increased in AD Brain

The combination of LPS-IS-HY demonstrated evidence of myelin injury in the adult rat brain (Zhan et al., 2015a). Thus, a search for evidence of myelin injury in AD compared to control brains was undertaken, and determined if LPS was associated with the myelin injury in AD brain.

**Figure 4:**
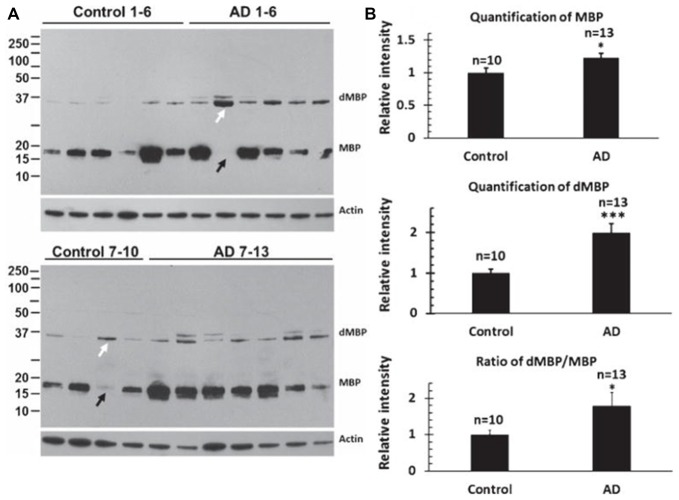
Assessing myelin amounts and myelin damage in AD and control brains. **(A)** Western blots for intact Myelin Basic Protein (MBP) and degraded Myelin Basic Protein (dMBP) show more dMBP in AD compared to control brains. Actin served as a lane loading control. **(B)** Quantification of the bands showed more MBP in AD compared to controls brains, more dMBP in AD compared to control brains, and a greater dMBP/MBP ratio in AD compared to control brains (*p < 0.05; ***p < 0.001). This Figure is from Zhan et al. (2015b). Reproduced with permission.

MBP and dMBP levels were examined in 13 AD brains and 10 control brains (Figure 4; Zhan et al., 2015b). dMBP was found in every AD brain, but not in every control aging brains (Figure 4A). Quantification showed more MBP in AD compared to control brains (Figure 4B), and much more dMBP in AD compared to control brains (Figure 4B). Indeed, the ratio of dMBP/MBP was greater in AD compared to control brains (Figure 4B; Zhan et al., 2015b). The antibody to dMBP stains a protein at 37 kDa which is higher in molecular weight compared to intact MBP. It was postulated that the antibody to dMBP probably detects some fragment of MBP (degraded MBP/dMBP) which complexes with other molecule(s) (Zhan et al., 2015b).

**Figure 5:**
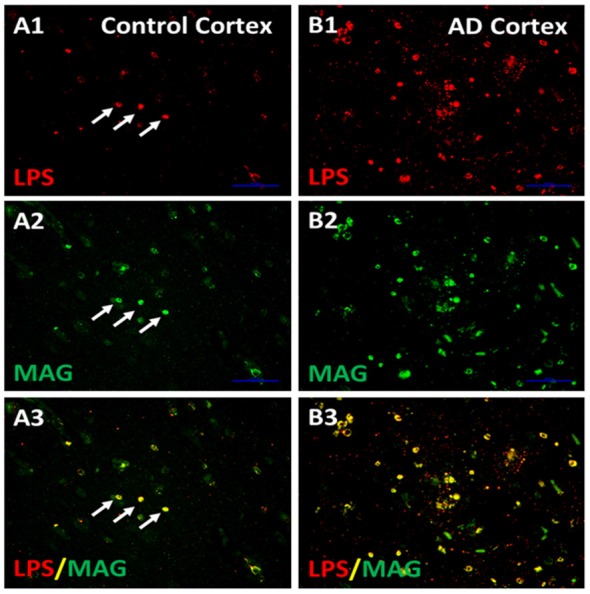
LPS co-localizes with MAG stained oligodendrocytes. There were more LPS stained cells in AD cortex compared to Control Cortex **(A1,B1)**. There were more MAG stained oligodendrocytes in in AD cortex compared to Control cortex **(A2,B2)**. Merging of images for LPS and MAG showed that LPS co-localized with MAG stained oligodendrocytes, and there were more LPS-MAG stained oligodendrocytes in AD cortex compared to control **(A3,B3)**. This is a supplementary figure from Zhan et al. (2016). Reproduced with permission.

The next studies determined whether LPS co-localized with MAG (myelin associated glycoprotein) stained oligodendrocytes in cortex (Figure 5; Zhan et al., 2016). There appeared to be more MAG stained oligodendrocytes in AD (Figure 5B2) compared to control cortex (Figure 5A2). There were more LPS stained cells in AD (Figure 5B1) compared to control (Figure 5A1) cortex. Most LPS stained cells in cortex co-localized with MAG stained oligodendrocytes (Figures 5A3,B3), with more LPS/MAG stained oligodendrocytes in AD (Figure 5B3) compared to control cortex (Figure 5A3; Zhan et al., 2016). These data show more oligodendrocytes in AD brain compared to control and could explain higher MBP levels in AD brain (Figure 4B). In addition, since LPS co-localizes with MAG stained oligodendrocytes, this suggests LPS could damage oligodendrocytes leading to increased dMBP seen in AD brain (Figure 4B). LPS acts on the TLR4 receptor to activate NFkB which increases cytokines that can damage oligodendrocytes and damage myelin proteins (Pang et al., 2003, 2010; Deng et al., 2008, 2014; Paintlia et al., 2008). Not shown here is an increased number of oligodendrocyte progenitor cells (OPCs) in AD compared to control brain (Zhan et al., 2016). The data suggests that LPS injures oligodendrocytes and myelin proteins including dMBP and MAG, and that this leads to proliferation of OPCs that differentiate into mature oligodendrocytes (Zhan et al., 2016).

**Figure 6:**
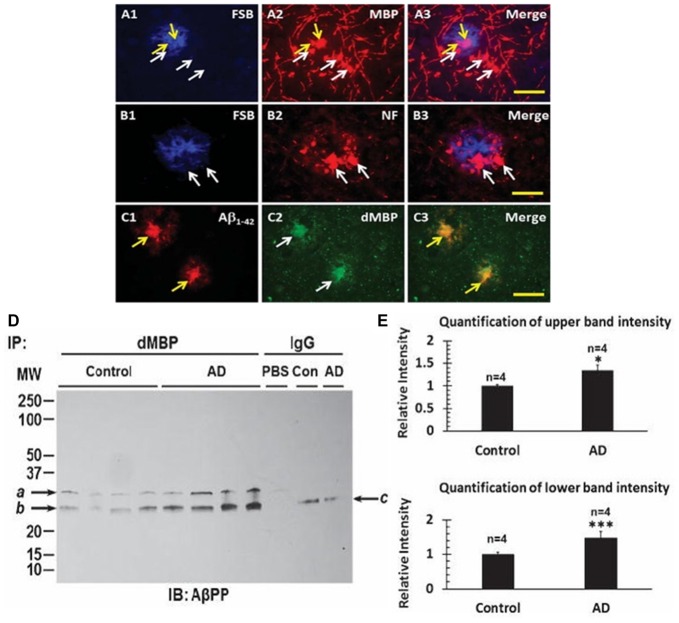
Myelin proteins in amyloid plaques of AD brains. FSB stained amyloid plaques **(A1,B1)** showed Myelin Basic Protein (MBP) around the plaques **(A2,A3)**, and Neurofilament (NF) protein around the plaques **(B2,B3)**. FSB did not co-localize with MBP **(A3)** or with NF **(B3)**. In contrast, dMBP formed aggregates **(C2)** similar in size to Aβ_1–42_ aggregates **(C1)**, with Aβ_1–42_ and dMBP being co-localized **(C3)**. FSB is (E, E)-1-fluoro-2, 5-bis (3-hydroxycarbonyl-4-hydroxy) styrylbenzene which is a Congo red derivative. Bar = 25 μm. **(D)** Immunoprecipitation of cortex with an antibody to dMBP followed by Western blotting for AβPP showed two bands in AD and control cortex, with greater amounts in the 4 AD subjects compared to the 4 Control subjects. **(E)** Quantification of the immunoprecipitation studies in **(D)** showed greater intensity of the upper and lower bands in the AD cortex compared to Control cortex (*P < 0.05; ***P < 0.001). This Figure is from Zhan et al. (2015b). Reproduced with permission.

With evidence of consistent myelin injury in AD brain, the localization of dMBP in AD brain was examined. Neither MBP (Figure 6A2) nor neurofilament protein (NF; Figure 6B2) co-localized with FSB ((E, E)-1-fluoro-2, 5-bis (3-hydroxycarbonyl-4-hydroxy) styrylbenzene, Figures 6A1,B1) stained amyloid plaques in AD brain (Figures 6A3,B3). However, Aβ_1–42_ (Figure 6C1) did co-localize with dMBP (Figure 6C2) in amyloid plaques in AD brain (Figure 6C3; Zhan et al., 2015b). These data were supported by biochemical findings suggesting dMBP directly bound AβPP and Aβ_1–42_ (Zhan et al., 2015b). Figure 6D shows that immunoprecipitation with an antibody to dMBP followed by Western blotting with an antibody to AβPP showed bands in AD and control brain (Figure 6D), but with greater levels in AD brain (Figure 6E; Zhan et al., 2015b). These data support the idea that LPS injures oligodendrocytes with the resultant dMBP binding to and being co-localized with Aβ within amyloid plaques in AD brain.

**Figure 7:**
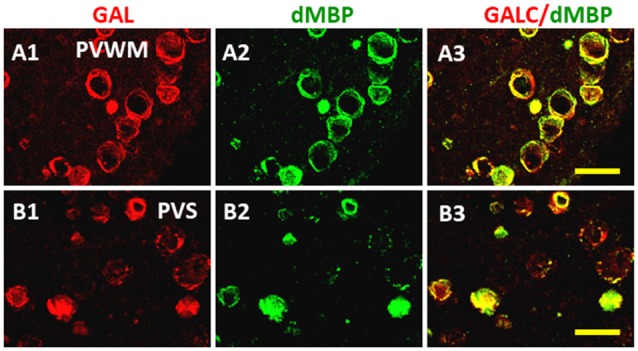
Localization of dMBP in AD brain in periventricular white matter (PVWM) and in the perivascular space of white matter (PVSR). The WM galactocerebroside (GALC) **(A1,B1)** co-localized with dMBP **(A2,B2)** in the PVWM **(A3)** and in PVSR **(B3)**. The immunofluorescence occurred in vesicles throughout the PVWM and PVSR. This Figure is from Zhan et al. (2014). Reproduced with permission.

The distribution of dMBP in WM of AD brain was also examined (Figure 7; Zhan et al., 2014). Using an antibody to galactocerebroside (GALC) as a marker for myelin, many GALC stained walls of vesicles in periventricular WM (PVWM; Figure 7A1) and the perivascular space (Figure 7B1) of AD brains were found (Zhan et al., 2014). These GALC stained vesicles were shown to co-localize with dMBP in both periventricular WM (Figures 7A2,A3) and in the perivascular space (Figures 7B2,B3) of AD brains. There were fewer vesicles in control brains in the periventricular WM (not shown). The above results show consistent myelin injury in both AD gray and WM (Zhan et al., 2015b).

## Proposed Model of LPS Induced Injury in AD Brain

**Figure 8:**
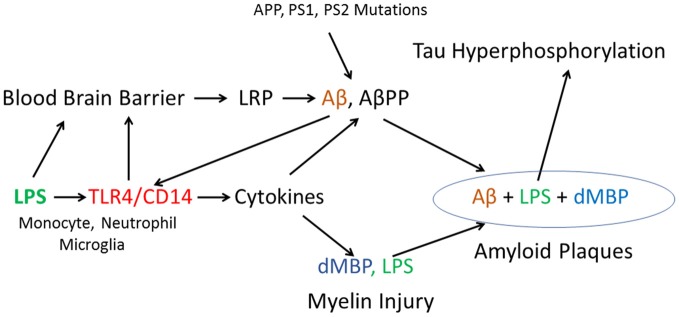
Proposed model of how LPS, in combination with other factors, might produce amyloid plaques, myelin injury and Tau hyperphosphorylation. In addition, the model includes a possible mechanism by which autosomal dominant mutations in AβPP and Presenilin could damage myelin via Aβ actions on TLR4/CD14 receptors to increase cytokines.

A tentative model of injury is shown in Figure 8 where LPS binds to TLR4/CD14 receptors on peripheral monocytes/macrophages, neutrophils and on brain microglia. TLR4/CD14 activation by LPS leads to NFκB mediated induction of cytokines including IL1, IL6 and TNF in monocytes/neutrophils in blood and from microglia in brain. Since LPS does not enter normal brain when given alone (Banks and Erickson, 2010; Banks et al., 2015), it is likely that other factors contribute to LPS entry into aging brain including ischemia, hypoxia, peripheral cytokines and other factors. Impaired blood brain barrier (BBB) and areas devoid of BBB might aid entry of LPS to the brain. Once LPS entered brain it would bind to TLR4/CD14 receptors on microglia which would activate NFκB mediated increases of intracerebral cytokines. Very high levels of cytokines produce myelin injury (see below). LPS induction of cytokines can also increase accumulation of AβPP and Aβ, which in turn can act on TLR4 to create a positive feedback loop to increase Aβ (Wu et al., 2015). LPS also acts on the BBB to decrease Aβ exit from brain (Banks et al., 2015; Figure 8). Aggregation of Aβ, AβPP, degraded myelin proteins (including dMBP) and LPS contributes to formation of amyloid plaques (see below; Figure 8). Finally, LPS is known to induce tau hyper phosphorylation (Kitazawa et al., 2005; Lee et al., 2010; Liu et al., 2016; Figure 8).

### Role of LPS

Gram-negative *E. coli* bacteria can synthesize extracellular amyloid (Zhao and Lukiw, 2015). This may be relevant since a recent RNAseq study demonstrated bacterial molecules in human brain, and showed the majority were associated with Gram-negative, LPS containing alpha Proteobacteria (Branton et al., 2013). Thus, the LPS found in AD brain could be derived from molecules of Gram-negative bacteria entering brain from the blood, from Gram-negative bacteria entering brain, or possibly from endogenous Gram-negative bacteria in brain.

LPS binds the TLR4/CD14 complex on peripheral monocytes/macrophages or brain microglia to activate NFκB and increase production of cytokines including IL1, IL6 and TNF (Ikeda et al., 1999; Rossol et al., 2011; Enkhbaatar et al., 2015). LPS down regulation of ADAM10 and upregulation of BACE-1/PS-1 may partially explain LPS induced increases β-AβPP and Aβ (Sheng et al., 2003; Zhao et al., 2014; Wu et al., 2015). LPS also increases Aβ levels in brain by acting at the blood brain barrier and impairing LRP which is responsible for Aβ efflux from brain (Erickson et al., 2012; Banks et al., 2015). LPS at high doses can damage the BBB (Banks et al., 2015) which could facilitate entry of LPS itself into brain. Alternatively, monocytes/macrophages may carry LPS into brain. LPS binding to TLR4 on endothelial cells also leads to cytokine release (Verma et al., 2006). LPS binds serum amyloid P and Aβ (de Haas et al., 2000), it binds MBP (Raziuddin and Morrison, 1981) and LPS promotes tau hyper phosphorylation (Kitazawa et al., 2005; Lee et al., 2010; Liu et al., 2016). Thus, LPS could contribute to all of the key neuropathological findings in AD brain including: amyloid plaques, myelin injury and tau hyperphosphorylation (Figure 8).

### Role of TLR4/CD14 Complex

Polymorphisms in TLR4 and CD14 have been associated with AD in some studies (Balistreri et al., 2008; Rodríguez-Rodríguez et al., 2008; Chen et al., 2012) and whole genome studies have implicated TLR4 in AD (Li et al., 2015). TLR4 levels are increased in AβPP transgenic AD mice and human AD brain and treatment of microglia with amyloid peptide increases IL6 and TNF in microglia and kills microglia via the TLR4 receptor (Walter et al., 2007). The microglial TLR4 receptor is required for recruitment of leukocytes into brain in response to intracranial injections of LPS (Zhou et al., 2006). CD14 binds amyloid peptide fibrils (Fassbender et al., 2004) and is a microglial receptor for phagocytosis of Aβ (Liu et al., 2005). Deletion of CD14 attenuates pathology in AD mice by decreasing inflammation (Reed-Geaghan et al., 2010). In the neonatal LPS/hypoxia model, LPS mediates death of oligodendrocytes via the TLR4 receptor (Lehnardt et al., 2002); and, LPS activated microglia can kill OPCs (Pang et al., 2010).

### Role of Cytokines in Myelin Injury

LPS-TLR4-NFκB mediated increase of cytokines is proposed to damage oligodendrocytes and myelin which leads to formation of myelin aggregates (Figure 8). TLR4 mediated increases of IL1, IL6 and TNF can kill mature oligodendrocytes, oligodendrocyte progenitors and damage myelin (Fan et al., 2009; Xie et al., 2016). Tumor necrosis factor alpha mediates lipopolysaccharide-induced microglial toxicity to developing oligodendrocytes when astrocytes are present (Selmaj and Raine, 1988; Li et al., 2008). Other cytokines are also induced by TLR4 activation and these might also play a role in injury.

### Damaged Myelin Interacts with Aβ

MBP directly binds AβPP and Aβ and inhibits Aβ fibrillary assembly via residues 54–64 in MBP (Liao et al., 2009, 2010; Kotarba et al., 2013). Pure MBP degrades AβPP and Aβ peptides, though degraded MBP lacking autolytic activity may not degrade Aβ_40_ or Aβ_42_ (Liao et al., 2009). A knockout mouse of MBP (bigenic Tg-5xFAD/MBP−/−) showed markedly decreased numbers of amyloid plaques and decreased insoluble Aβ (Ou-Yang and Van Nostrand, 2013). These findings suggest degraded MBP (dMBP) might play a role in amyloid plaque formation. Axonal/myelin injury results in formation of myelin aggregates, and degraded MBP binds AβPP and Aβ peptides (Liao et al., 2009, 2010; Kotarba et al., 2013), and along with LPS and other molecules leads to the formation of amyloid plaques.

### Could the LPS Model Relate to Familial AD?

Familial AD is due to mutations in AβPP, presenilin-1 and presenilin-2, and these mutations result in an increase of brain and blood Aβ. It is still debated whether soluble or insoluble Aβ are important, and how they relate to tau hyper phosphorylation. Several groups have proposed that Aβ actions on the TLR4/CD14 complex could be important in the pathogenesis of AD (Figure 8). Aβ binding to TLR4/CD14 would increase cytokines which would lead to further increases of Aβ and to myelin injury and production of dMBP (Figure 8). This could help explain the association of WMH/myelin injury with familial AD (Lee et al., 2016).

**Figure 9:**
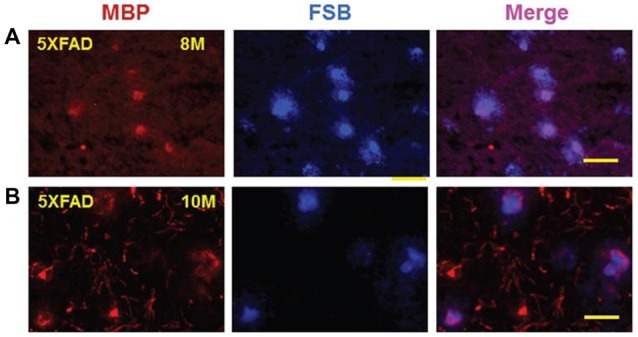
Myelin basic protein (MBP) colocalizes with FSB ((E, E)-1-fluoro-2, 5-bis (3-hydroxycarbonyl-4-hydroxy) styrylbenzene) stained amyloid plaques in cortex of 5XFAD mice. MBP stained aggregates in cortex of 8-month-old **(A)** and 10-month-old **(B)** 5XFAD mice co-localized with FSB stained amyloid plaques. Bar = 50 μm. This Figure is from Zhan et al. (2015a). Reproduced with permission.

To begin to address this, studies were done to determine whether a mouse model of AD which contained 3 AβPP mutations and 2 presenilin-1 mutations (5XFAD mouse) had myelin aggregates associated with its amyloid plaques. Indeed, at 8 months (Figure 9A) and 10 months of age (Figure 9B) the 5XFAD mouse showed that MBP stained myelin aggregates co-localized with FSB stained amyloid plaques (Zhan et al., 2015a). Though it is not known how the myelin is damaged, it is possible that Aβ activation of TLR4 could lead to myelin injury as shown in Figure 8. That is, both LPS and Aβ are ligands for TLR4/CD14 (Erridge, 2010) and thus both could contribute to myelin injury. This conclusion supports recent imaging studies that found WMH to be a core feature of autosomal dominant familial AD (Lee et al., 2016). Thus, sporadic WMH could be a result of the actions of LPS-TLR4 mediated injury and/or Aβ-TLR4 mediated injury to myelin possibly in combination with ischemia/hypoxia.

### Caveats to the Proposed Model

Though the above discussion focuses on LPS, other Gram-negative molecules are found in AD brain (Zhan et al., 2016). The significance of these to AD pathogenesis is not clear, but could be important. The source of LPS and other bacterial molecules in brain is not clear, so that exogenous infections vs. some source within the body or the brain must be resolved. Since LPS is in AD brain, perhaps molecules from other classes of infectious agents might also be relevant in subgroups of AD cases that might be mediated via other Toll-like receptors.

## Conclusion

It is hypothesized that LPS, in combination with other factors, leads to amyloid plaques, myelin injury and tau hyperphosphorylation in AD brain. Since the presence of LPS in human AD brain has been confirmed in different laboratories, treatment and prevention targets for sporadic AD could include LPS, TLR4/CD14 receptors, and Gram-negative bacteria. A vaccine against LPS to prevent AD could be considered if future studies continue to support a role of LPS in AD.

## Author Contributions

XZ, BS and FRS designed the studies, analyzed the data, interpreted the data and wrote the manuscript and gave approval for publication of this version of the manuscript. They agree to be accountable for all aspects of the work in ensuring that questions related to the accuracy and integrity of any part of the work were appropriately investigated and resolved.

## Conflict of Interest Statement

The authors declare that the research was conducted in the absence of any commercial or financial relationships that could be construed as a potential conflict of interest.

## References

[B1] Abbayya K. Puthanakar N. Y. Naduwinmani S. Chidambar Y. S. (2015). Association between Periodontitis and Alzheimer’s Disease. N. Am. J. Med. Sci. 7, 241–246. 10.4103/1947-2714.159325 26199919 PMC4488989

[B2] Alzheimer A. Förstl H. Levy R. (1991). On certain peculiar diseases of old age. Hist. Psychiatry 2, 71–101. 10.1177/0957154X9100200505 11622845

[B3] Bai Z. Stamova B. Xu H. Ander B. P. Wang J. Jickling G. C.. (2014). Distinctive RNA expression profiles in blood associated with Alzheimer disease after accounting for white matter hyperintensities. Alzheimer Dis. Assoc. Disord. 28, 226–233. 10.1097/WAD.0000000000000022 24731980 PMC4139468

[B4] Balistreri C. R. Grimaldi M. P. Chiappelli M. Licastro F. Castiglia L. Listi F.. (2008). Association between the polymorphisms of TLR4 and CD14 genes and Alzheimer’s disease. Curr. Pharm. Des. 14, 2672–2677. 10.2174/138161208786264089 19006850

[B5] Banks W. A. Erickson M. A. (2010). The blood-brain barrier and immune function and dysfunction. Neurobiol. Dis. 37, 26–32. 10.1016/j.nbd.2009.07.031 19664708

[B6] Banks W. A. Gray A. M. Erickson M. A. Salameh T. S. Damodarasamy M. Sheibani N.. (2015). Lipopolysaccharide-induced blood-brain barrier disruption: roles of cyclooxygenase, oxidative stress, neuroinflammation, and elements of the neurovascular unit. J. Neuroinflammation 12:223. 10.1186/s12974-015-0434-1 26608623 PMC4660627

[B7] Bartzokis G. (2011). Alzheimer’s disease as homeostatic responses to age-related myelin breakdown. Neurobiol. Aging 32, 1341–1371. 10.1016/j.neurobiolaging.2009.08.007 19775776 PMC3128664

[B8] Belkacemi A. Ramassamy C. (2012). Time sequence of oxidative stress in the brain from transgenic mouse models of Alzheimer’s disease related to the amyloid-β cascade. Free Radic. Biol. Med. 52, 593–600. 10.1016/j.freeradbiomed.2011.11.020 22172527

[B10] Bertram L. Lill C. M. Tanzi R. E. (2010). The genetics of Alzheimer disease: back to the future. Neuron 68, 270–281. 10.1016/j.neuron.2010.10.013 20955934

[B9] Bertram L. Tanzi R. E. (2012). The genetics of Alzheimer’s disease. Prog. Mol. Biol. Transl. Sci. 107, 79–100. 10.1016/B978-0-12-385883-2.00008-4 22482448

[B11] Bester J. Soma P. Kell D. B. Pretorius E. (2015). Viscoelastic and ultrastructural characteristics of whole blood and plasma in Alzheimer-type dementia and the possible role of bacterial lipopolysaccharides (LPS). Oncotarget 6, 35284–35303. 10.18632/oncotarget.6074 26462180 PMC4742105

[B13] Blanco L. P. Evans M. L. Smith D. R. Badtke M. P. Chapman M. R. (2012). Diversity, biogenesis and function of microbial amyloids. Trends Microbiol. 20, 66–73. 10.1016/j.tim.2011.11.005 22197327 PMC3278576

[B14] Branton W. G. Ellestad K. K. Maingat F. Wheatley B. M. Rud E. Warren R. L.. (2013). Brain microbial populations in HIV/AIDS: α-proteobacteria predominate independent of host immune status. PLoS One 8:e54673. 10.1371/journal.pone.0054673 23355888 PMC3552853

[B15] Branton W. G. Lu J. Q. Surette M. G. Holt R. A. Lind J. Laman J. D.. (2016). Brain microbiota disruption within inflammatory demyelinating lesions in multiple sclerosis. Sci. Rep. 6:37344. 10.1038/srep37344 27892518 PMC5125007

[B16] Brickman A. M. Honig L. S. Scarmeas N. Tatarina O. Sanders L. Albert M. S.. (2008). Measuring cerebral atrophy and white matter hyperintensity burden to predict the rate of cognitive decline in Alzheimer disease. Arch. Neurol. 65, 1202–1208. 10.1001/archneur.65.9.1202 18779424 PMC2629007

[B17] Buggia-Prevot V. Sevalle J. Rossner S. Checler F. (2008). NFκB-dependent control of BACE1 promoter transactivation by Aβ42. J. Biol. Chem. 283, 10037–10047. 10.1074/jbc.M706579200 18263584

[B18] Butchart J. Holmes C. (2012). Systemic and central immunity in Alzheimer’s disease: therapeutic implications. CNS Neurosci. Ther. 18, 64–76. 10.1111/j.1755-5949.2011.00245.x 22070806 PMC6493531

[B19] Cattaneo A. Cattane N. Galluzzi S. Provasi S. Lopizzo N. Festari C.. (2017). Association of brain amyloidosis with pro-inflammatory gut bacterial taxa and peripheral inflammation markers in cognitively impaired elderly. Neurobiol. Aging 49, 60–68. 10.1016/j.neurobiolaging.2016.08.019 27776263

[B20] Cavanaugh S. E. Pippin J. J. Barnard N. D. (2014). Animal models of Alzheimer disease: historical pitfalls and a path forward. ALTEX 31, 279–302. 10.14573/altex.1310071 24793844

[B21] Chen C. K. Wu Y. T. Chang Y. C. (2017). Association between chronic periodontitis and the risk of Alzheimer’s disease: a retrospective, population-based, matched-cohort study. Alzheimers. Res. Ther. 9:56. 10.1186/s13195-017-0282-6 28784164 PMC5547465

[B22] Chen Y. C. Yip P. K. Huang Y. L. Sun Y. Wen L. L. Chu Y. M.. (2012). Sequence variants of toll like receptor 4 and late-onset Alzheimer’s disease. PLoS One 7:e50771. 10.1371/journal.pone.0050771 23272070 PMC3525588

[B23] Civitelli L. Marcocci M. E. Celestino I. Piacentini R. Garaci E. Grassi C.. (2015). Herpes simplex virus type 1 infection in neurons leads to production and nuclear localization of APP intracellular domain (AICD): implications for Alzheimer’s disease pathogenesis. J. Neurovirol. 21, 480–490. 10.1007/s13365-015-0344-0 25925093

[B24] Couttas T. A. Kain N. Suchowerska A. K. Quek L. E. Turner N. Fath T.. (2016). Loss of ceramide synthase 2 activity, necessary for myelin biosynthesis, precedes tau pathology in the cortical pathogenesis of Alzheimer’s disease. Neurobiol. Aging 43, 89–100. 10.1016/j.neurobiolaging.2016.03.027 27255818

[B25] Crehan H. Holton P. Wray S. Pocock J. Guerreiro R. Hardy J. (2012). Complement receptor 1 (CR1) and Alzheimer’s disease. Immunobiology 217, 244–250. 10.1016/j.imbio.2011.07.017 21840620

[B26] de Craen A. J. Gussekloo J. Vrijsen B. Westendorp R. G. (2005). Meta-analysis of nonsteroidal antiinflammatory drug use and risk of dementia. Am. J. Epidemiol. 161, 114–120. 10.1093/aje/kwi029 15632261

[B27] de Haas C. J. van Leeuwen E. M. van Bommel T. Verhoef J. van Kessel K. P. van Strijp J. A. (2000). Serum amyloid P component bound to gram-negative bacteria prevents lipopolysaccharide-mediated classical pathway complement activation. Infect. Immun. 68, 1753–1759. 10.1128/iai.68.4.1753-1759.2000 10722560 PMC97344

[B28] de la Torre J. C. (2009). Cerebrovascular and cardiovascular pathology in Alzheimer’s disease. Int. Rev. Neurobiol. 84, 35–48. 10.1016/s0074-7742(09)00403-6 19501712

[B29] Deng W. Pleasure J. Pleasure D. (2008). Progress in periventricular leukomalacia. Arch. Neurol. 65, 1291–1295. 10.1001/archneur.65.10.1291 18852342 PMC2898886

[B30] Deng Y. Xie D. Fang M. Zhu G. Chen C. Zeng H.. (2014). Astrocyte-derived proinflammatory cytokines induce hypomyelination in the periventricular white matter in the hypoxic neonatal brain. PLoS One 9:e87420. 10.1371/journal.pone.0087420 24498101 PMC3909103

[B31] Desai M. K. Mastrangelo M. A. Ryan D. A. Sudol K. L. Narrow W. C. Bowers W. J. (2010). Early oligodendrocyte/myelin pathology in Alzheimer’s disease mice constitutes a novel therapeutic target. Am. J. Pathol. 177, 1422–1435. 10.2353/ajpath.2010.100087 20696774 PMC2928974

[B32] Dunn N. Mullee M. Perry V. H. Holmes C. (2005). Association between dementia and infectious disease: evidence from a case-control study. Alzheimer Dis. Assoc. Disord. 19, 91–94. 10.1097/01.wad.0000165511.52746.1f 15942327

[B33] Emery D. C. Shoemark D. K. Batstone T. E. Waterfall C. M. Coghill J. A. Cerajewska T. L.. (2017). 16S rRNA next generation sequencing analysis shows bacteria in Alzheimer’s post-mortem brain. Front. Aging Neurosci. 9:195. 10.3389/fnagi.2017.00195 28676754 PMC5476743

[B34] Engelhart M. J. Geerlings M. I. Meijer J. Kiliaan A. Ruitenberg A. van Swieten J. C.. (2004). Inflammatory proteins in plasma and the risk of dementia: the rotterdam study. Arch. Neurol. 61, 668–672. 10.1001/archneur.61.5.668 15148142

[B35] Englund E. (1998). Neuropathology of white matter changes in Alzheimer’s disease and vascular dementia. Dement. Geriatr. Cogn. Disord. 9, 6–12. 10.1159/000051183 9716238

[B36] Enkhbaatar P. Nelson C. Salsbury J. R. Carmical J. R. Torres K. E. Herndon D.. (2015). Comparison of gene expression by sheep and human blood stimulated with the TLR4 agonists lipopolysaccharide and monophosphoryl lipid A. PLoS One 10:e0144345. 10.1371/journal.pone.0144345 26640957 PMC4671644

[B37] Erickson M. A. Hartvigson P. E. Morofuji Y. Owen J. B. Butterfield D. A. Banks W. A. (2012). Lipopolysaccharide impairs amyloid β efflux from brain: altered vascular sequestration, cerebrospinal fluid reabsorption, peripheral clearance and transporter function at the blood-brain barrier. J. Neuroinflammation 9:150. 10.1186/1742-2094-9-150 22747709 PMC3410805

[B38] Erridge C. (2010). Endogenous ligands of TLR2 and TLR4: agonists or assistants? J. Leukoc. Biol. 87, 989–999. 10.1189/jlb.1209775 20179153

[B39] Fan L. W. Mitchell H. J. Tien L. T. Rhodes P. G. Cai Z. (2009). Interleukin-1β-induced brain injury in the neonatal rat can be ameliorated by α-phenyl-n-tert-butyl-nitrone. Exp. Neurol. 220, 143–153. 10.1016/j.expneurol.2009.08.003 19682987 PMC2761495

[B40] Fassbender K. Walter S. Kühl S. Landmann R. Ishii K. Bertsch T.. (2004). The LPS receptor (CD14) links innate immunity with Alzheimer’s disease. FASEB J. 18, 203–205. 10.1096/fj.03-0364fje 14597556

[B41] Gaultier A. Wu X. Le Moan N. Takimoto S. Mukandala G. Akassoglou K.. (2009). Low-density lipoprotein receptor-related protein 1 is an essential receptor for myelin phagocytosis. J. Cell Sci. 122, 1155–1162. 10.1242/jcs.040717 19299462 PMC2714439

[B42] Goate A. Hardy J. (2012). Twenty years of Alzheimer’s disease-causing mutations. J. Neurochem. 120, 3–8. 10.1111/j.1471-4159.2011.07575.x 22122678

[B43] Grammas P. (2011). Neurovascular dysfunction, inflammation and endothelial activation: implications for the pathogenesis of Alzheimer’s disease. J. Neuroinflammation 8:26. 10.1186/1742-2094-8-26 21439035 PMC3072921

[B44] Guglielmotto M. Monteleone D. Boido M. Piras A. Giliberto L. Borghi R.. (2012). Aβ1–42-mediated down-regulation of Uch-L1 is dependent on NF-κB activation and impaired BACE1 lysosomal degradation. Aging Cell 11, 834–844. 10.1111/j.1474-9726.2012.00854.x 22726800

[B45] Guglielmotto M. Tamagno E. Danni O. (2009). Oxidative stress and hypoxia contribute to Alzheimer’s disease pathogenesis: two sides of the same coin. ScientificWorldJournal 9, 781–791. 10.1100/tsw.2009.93 19705038 PMC5823176

[B46] Hagberg H. Peebles D. Mallard C. (2002). Models of white matter injury: comparison of infectious, hypoxic-ischemic, and excitotoxic insults. Ment. Retard. Dev. Disabil. Res. Rev. 8, 30–38. 10.1002/mrdd.10007 11921384

[B47] Hall A. M. Roberson E. D. (2012). Mouse models of Alzheimer’s disease. Brain Res. Bull. 88, 3–12. 10.1016/j.brainresbull.2011.11.017 22142973 PMC3546481

[B48] Hammond C. J. Hallock L. R. Howanski R. J. Appelt D. M. Little C. S. Balin B. J. (2010). Immunohistological detection of Chlamydia pneumoniae in the Alzheimer’s disease brain. BMC Neurosci. 11:121. 10.1186/1471-2202-11-121 20863379 PMC2949767

[B49] Heneka M. T. Carson M. J. El Khoury J. Landreth G. E. Brosseron F. Feinstein D. L.. (2015). Neuroinflammation in Alzheimer’s disease. Lancet Neurol. 14, 388–405. 10.1016/S1474-4422(15)70016-5 25792098 PMC5909703

[B50] Herholz K. (2010). Cerebral glucose metabolism in preclinical and prodromal Alzheimer’s disease. Expert Rev. Neurother. 10, 1667–1673. 10.1586/ern.10.136 20977325

[B51] Herrup K. (2015). The case for rejecting the amyloid cascade hypothesis. Nat. Neurosci. 18, 794–799. 10.1038/nn.4017 26007212

[B52] Hill J. M. Lukiw W. J. (2015). Microbial-generated amyloids and Alzheimer’s disease (AD). Front. Aging Neurosci. 7:9. 10.3389/fnagi.2015.00009 25713531 PMC4322713

[B53] Horiuchi M. Maezawa I. Itoh A. Wakayama K. Jin L. W. Itoh T.. (2012). Amyloid β1–42 oligomer inhibits myelin sheet formation *in vitro*. Neurobiol. Aging 33, 499–509. 10.1016/j.neurobiolaging.2010.05.007 20594620 PMC3013291

[B54] Huang Y. Mucke L. (2012). Alzheimer mechanisms and therapeutic strategies. Cell 148, 1204–1222. 10.1016/j.cell.2012.02.040 22424230 PMC3319071

[B55] Hunt A. Schönknecht P. Henze M. Seidl U. Haberkorn U. Schröder J. (2007). Reduced cerebral glucose metabolism in patients at risk for Alzheimer’s disease. Psychiatry Res. 155, 147–154. 10.1016/j.pscychresns.2006.12.003 17524628

[B56] Iadecola C. (2004). Neurovascular regulation in the normal brain and in Alzheimer’s disease. Nat. Rev. Neurosci. 5, 347–360. 10.1038/nrn1387 15100718

[B57] Iadecola C. (2013). The pathobiology of vascular dementia. Neuron 80, 844–866. 10.1016/j.neuron.2013.10.008 24267647 PMC3842016

[B58] Iadecola C. Zhang F. Niwa K. Eckman C. Turner S. K. Fischer E.. (1999). SOD1 rescues cerebral endothelial dysfunction in mice overexpressing amyloid precursor protein. Nat. Neurosci. 2, 157–161. 10.1038/5715 10195200

[B59] Ikeda M. Hamada K. Sumitomo N. Okamoto H. Sakakibara B. (1999). Serum amyloid A, cytokines, and corticosterone responses in germfree and conventional mice after lipopolysaccharide injection. Biosci. Biotechnol. Biochem. 63, 1006–1010. 10.1271/bbb.63.1006 10427685

[B60] Imbimbo B. P. (2009). An update on the efficacy of non-steroidal anti-inflammatory drugs in Alzheimer’s disease. Expert Opin. Investig. Drugs 18, 1147–1168. 10.1517/13543780903066780 19589092

[B61] Imbimbo B. P. Solfrizzi V. Panza F. (2010). Are NSAIDs useful to treat Alzheimer’s disease or mild cognitive impairment? Front. Aging Neurosci. 2:19. 10.3389/fnagi.2010.00019 20725517 PMC2912027

[B62] Ishida N. Ishihara Y. Ishida K. Tada H. Funaki-Kato Y. Hagiwara M.. (2017). Periodontitis induced by bacterial infection exacerbates features of Alzheimer’s disease in transgenic mice. NPJ Aging Mech. Dis. 3:15. 10.1038/s41514-017-0015-x 29134111 PMC5673943

[B63] Kamer A. R. Pirraglia E. Tsui W. Rusinek H. Vallabhajosula S. Mosconi L.. (2015). Periodontal disease associates with higher brain amyloid load in normal elderly. Neurobiol. Aging 36, 627–633. 10.1016/j.neurobiolaging.2014.10.038 25491073 PMC4399973

[B64] Kandel B. M. Avants B. B. Gee J. C. McMillan C. T. Erus G. Doshi J.. (2016). White matter hyperintensities are more highly associated with preclinical Alzheimer’s disease than imaging and cognitive markers of neurodegeneration. Alzheimers Dement. 4, 18–27. 10.1016/j.dadm.2016.03.001 27489875 PMC4950175

[B65] Karch C. M. Goate A. M. (2015). Alzheimer’s disease risk genes and mechanisms of disease pathogenesis. Biol. Psychiatry 77, 43–51. 10.1016/j.biopsych.2014.05.006 24951455 PMC4234692

[B66] Kitazawa M. Oddo S. Yamasaki T. R. Green K. N. LaFerla F. M. (2005). Lipopolysaccharide-induced inflammation exacerbates tau pathology by a cyclin-dependent kinase 5-mediated pathway in a transgenic model of Alzheimer’s disease. J. Neurosci. 25, 8843–8853. 10.1523/JNEUROSCI.2868-05.2005 16192374 PMC6725603

[B67] Kotarba A. E. Aucoin D. Hoos M. D. Smith S. O. Van Nostrand W. E. (2013). Fine mapping of the amyloid β-protein binding site on myelin basic protein. Biochemistry 52, 2565–2573. 10.1021/bi4001936 23510371 PMC3640357

[B68] Kountouras J. Boziki M. Gavalas E. Zavos C. Deretzi G. Grigoriadis N.. (2009). Increased cerebrospinal fluid Helicobacter pylori antibody in Alzheimer’s disease. Int. J. Neurosci. 119, 765–777. 10.1080/00207450902782083 19326283

[B69] Kuo H. K. Yen C. J. Chang C. H. Kuo C. K. Chen J. H. Sorond F. (2005). Relation of C-reactive protein to stroke, cognitive disorders and depression in the general population: systematic review and meta-analysis. Lancet Neurol. 4, 371–380. 10.1016/s1474-4422(05)70099-5 15907742

[B70] Lee D. C. Rizer J. Selenica M. L. Reid P. Kraft C. Johnson A.. (2010). LPS- induced inflammation exacerbates phospho-tau pathology in rTg4510 mice. J. Neuroinflammation 7:56. 10.1186/1742-2094-7-56 20846376 PMC2949628

[B71] Lee S. Viqar F. Zimmerman M. E. Narkhede A. Tosto G. Benzinger T. L.. (2016). White matter hyperintensities are a core feature of Alzheimer’s disease: evidence from the Dominantly Inherited Alzheimer Network. Ann. Neurol. 79, 929–939. 10.1002/ana.24647 27016429 PMC4884146

[B72] Lehnardt S. Lachance C. Patrizi S. Lefebvre S. Follett P. L. Jensen F. E.. (2002). The toll-like receptor TLR4 is necessary for lipopolysaccharide-induced oligodendrocyte injury in the CNS. J. Neurosci. 22, 2478–2486. 11923412 10.1523/JNEUROSCI.22-07-02478.2002 PMC6758325

[B73] Lehnardt S. Massillon L. Follett P. Jensen F. E. Ratan R. Rosenberg P. A.. (2003). Activation of innate immunity in the CNS triggers neurodegeneration through a Toll-like receptor 4-dependent pathway. Proc. Natl. Acad. Sci. U S A 100, 8514–8519. 10.1073/pnas.1432609100 12824464 PMC166260

[B75] Li X. Long J. He T. Belshaw R. Scott J. (2015). Integrated genomic approaches identify major pathways and upstream regulators in late onset alzheimer’s disease. Sci. Rep. 5:12393. 10.1038/srep12393 26202100 PMC4511863

[B74] Li J. Ramenaden E. R. Peng J. Koito H. Volpe J. J. Rosenberg P. A. (2008). Tumor necrosis factor α mediates lipopolysaccharide-induced microglial toxicity to developing oligodendrocytes when astrocytes are present. J. Neurosci. 28, 5321–5330. 10.1523/JNEUROSCI.3995-07.2008 18480288 PMC2677805

[B76] Liao M. C. Ahmed M. Smith S. O. Van Nostrand W. E. (2009). Degradation of amyloid β protein by purified myelin basic protein. J. Biol. Chem. 284, 28917–28925. 10.1074/jbc.M109.050856 19692707 PMC2781437

[B77] Liao M. C. Hoos M. D. Aucoin D. Ahmed M. Davis J. Smith S. O.. (2010). N-terminal domain of myelin basic protein inhibits amyloid β-protein fibril assembly. J. Biol. Chem. 285, 35590–35598. 10.1074/jbc.M110.169599 20807757 PMC2975183

[B79] Liu Y. Walter S. Stagi M. Cherny D. Letiembre M. Schulz-Schaeffer W.. (2005). LPS receptor (CD14): a receptor for phagocytosis of Alzheimer’s amyloid peptide. Brain 128, 1778–1789. 10.1093/brain/awh531 15857927

[B78] Liu J. Wang D. Li S. Q. Yu Y. Ye R. D. (2016). Suppression of LPS-induced tau hyperphosphorylation by serum amyloid A. J. Neuroinflammation 13:28. 10.1186/s12974-016-0493-y 26838764 PMC4736117

[B80] Lovheim H. Olsson J. Weidung B. Johansson A. Eriksson S. Hallmans G.. (2018). Interaction between cytomegalovirus and herpes simplex virus type 1 associated with the risk of Alzheimer’s disease development. J. Alzheimers Dis. 61, 939–945. 10.3233/JAD-161305 29254081

[B81] Marlatt M. W. Lucassen P. J. Perry G. Smith M. A. Zhu X. (2008). Alzheimer’s disease: cerebrovascular dysfunction, oxidative stress, and advanced clinical therapies. J. Alzheimers Dis. 15, 199–210. 10.3233/jad-2008-15206 18953109 PMC2774209

[B82] McGeer P. L. Schulzer M. McGeer E. G. (1996). Arthritis and anti-inflammatory agents as possible protective factors for Alzheimer’s disease: a review of 17 epidemiologic studies. Neurology 47, 425–432. 10.1212/WNL.47.2.425 8757015

[B83] Meyer E. P. Ulmann-Schuler A. Staufenbiel M. Krucker T. (2008). Altered morphology and 3D architecture of brain vasculature in a mouse model for Alzheimer’s disease. Proc. Natl. Acad. Sci. U S A 105, 3587–3592. 10.1073/pnas.0709788105 18305170 PMC2265182

[B84] Miklossy J. (1993). Alzheimer’s disease—a spirochetosis? Neuroreport 4, 841–848. 10.1097/00001756-199307000-00002 8369471

[B85] Miklossy J. Kasas S. Janzer R. C. Ardizzoni F. Van der Loos H. (1994). Further ultrastructural evidence that spirochaetes may play a role in the aetiology of Alzheimer’s disease. Neuroreport 5, 1201–1204. 10.1097/00001756-199406020-00010 7919164

[B86] Miklossy J. Khalili K. Gern L. Ericson R. L. Darekar P. Bolle L.. (2004). Borrelia burgdorferi persists in the brain in chronic lyme neuroborreliosis and may be associated with Alzheimer disease. J. Alzheimers Dis. 6, 639–649; discussion 673–681. 10.3233/jad-2004-6608 15665404

[B87] Minter M. R. Zhang C. Leone V. Ringus D. L. Zhang X. Oyler-Castrillo P.. (2016). Antibiotic-induced perturbations in gut microbial diversity influences neuro-inflammation and amyloidosis in a murine model of Alzheimer’s disease. Sci. Rep. 6:30028. 10.1038/srep30028 27443609 PMC4956742

[B88] Mitew S. Kirkcaldie M. T. Halliday G. M. Shepherd C. E. Vickers J. C. Dickson T. C. (2010). Focal demyelination in Alzheimer’s disease and transgenic mouse models. Acta Neuropathol. 119, 567–577. 10.1007/s00401-010-0657-2 20198482

[B89] Möller H. J. Graeber M. B. (1998). The case described by Alois Alzheimer in 1911. Historical and conceptual perspectives based on the clinical record and neurohistological sections. Eur. Arch. Psychiatry Clin. Neurosci. 248, 111–122. 10.1007/s004060050027 9728729

[B500] Montagne A. Barnes S. R. Sweeney M. D. Halliday M. R. Sagare A. P. Zhao Z.. (2015). Blood-brain barrier breakdown in the aging human hippocampus. Neuron 85, 296–302. 10.1016/j.neuron.2014.12.032 25611508 PMC4350773

[B90] Montagne A. Zhao Z. Zlokovic B. V. (2017). Alzheimer’s disease: a matter of blood-brain barrier dysfunction? J. Exp. Med. 214, 3151–3169. 10.1084/jem.20171406 29061693 PMC5679168

[B91] Noble J. M. Scarmeas N. Celenti R. S. Elkind M. S. Wright C. B. Schupf N.. (2014). Serum IgG antibody levels to periodontal microbiota are associated with incident Alzheimer disease. PLoS One 9:e114959. 10.1371/journal.pone.0114959 25522313 PMC4270775

[B92] O’Brien R. J. Wong P. C. (2011). Amyloid precursor protein processing and Alzheimer’s disease. Annu. Rev. Neurosci. 34, 185–204. 10.1146/annurev-neuro-061010-113613 21456963 PMC3174086

[B93] Olsen I. Singhrao S. K. (2015). Can oral infection be a risk factor for Alzheimer’s disease? J. Oral. Microbiol. 7:29143. 10.3402/jom.v7.29143 26385886 PMC4575419

[B94] Ou-Yang M. H. Van Nostrand W. E. (2013). The absence of myelin basic protein promotes neuroinflammation and reduces amyloid β-protein accumulation in Tg-5xFAD mice. J. Neuroinflammation 10:134. 10.1186/1742-2094-10-134 24188129 PMC4228351

[B95] Paintlia M. K. Paintlia A. S. Contreras M. A. Singh I. Singh A. K. (2008). Lipopolysaccharide-induced peroxisomal dysfunction exacerbates cerebral white matter injury: attenuation by N-acetyl cysteine. Exp. Neurol. 210, 560–576. 10.1016/j.expneurol.2007.12.011 18291369 PMC2673813

[B96] Pang Y. Cai Z. Rhodes P. G. (2003). Disturbance of oligodendrocyte development, hypomyelination and white matter injury in the neonatal rat brain after intracerebral injection of lipopolysaccharide. Dev. Brain Res. 140, 205–214. 10.1016/s0165-3806(02)00606-5 12586426

[B97] Pang Y. Campbell L. Zheng B. Fan L. Cai Z. Rhodes P. (2010). Lipopolysaccharide-activated microglia induce death of oligodendrocyte progenitor cells and impede their development. Neuroscience 166, 464–475. 10.1016/j.neuroscience.2009.12.040 20035837

[B98] Papuc E. Kurys-Denis E. Krupski W. Tatara M. Rejdak K. (2015). Can antibodies against glial derived antigens be early biomarkers of hippocampal demyelination and memory loss in Alzheimer’s disease? J. Alzheimers Dis. 48, 115–121. 10.3233/JAD-150309 26401933

[B99] Pisa D. Alonso R. Juarranz A. Rábano A. Carrasco L. (2015). Direct visualization of fungal infection in brains from patients with Alzheimer’s disease. J. Alzheimers Dis. 43, 613–624. 10.3233/JAD-141386 25125470

[B100] Poole S. Singhrao S. K. Kesavalu L. Curtis M. A. Crean S. (2013). Determining the presence of periodontopathic virulence factors in short-term postmortem Alzheimer’s disease brain tissue. J. Alzheimers Dis. 36, 665–677. 10.3233/JAD-121918 23666172

[B101] Potgieter M. Bester J. Kell D. B. Pretorius E. (2015). The dormant blood microbiome in chronic, inflammatory diseases. FEMS Microbiol. Rev. 39, 567–591. 10.1093/femsre/fuv013 25940667 PMC4487407

[B102] Rahkonen T. Luukkainen-Markkula R. Paanila S. Sivenius J. Sulkava R. (2000a). Delirium episode as a sign of undetected dementia among community dwelling elderly subjects: a 2 year follow up study. J. Neurol. Neurosurg. Psychiatry 69, 519–521. 10.1136/jnnp.69.4.519 10990515 PMC1737142

[B103] Rahkonen T. Mäkelä H. Paanila S. Halonen P. Sivenius J. Sulkava R. (2000b). Delirium in elderly people without severe predisposing disorders: etiology and 1-year prognosis after discharge. Int. Psychogeriatr. 12, 473–481. 10.1017/s1041610200006591 11263714

[B104] Raziuddin S. Morrison D. C. (1981). Binding of bacterial endotoxin (LPS) to encephalitogenic myelin basic protein and modulation of characteristic biologic activities of LPS. J. Immunol. 126, 1030–1035. 6161956

[B105] Reed-Geaghan E. G. Reed Q. W. Cramer P. E. Landreth G. E. (2010). Deletion of CD14 attenuates Alzheimer’s disease pathology by influencing the brain’s inflammatory milieu. J. Neurosci. 30, 15369–15373. 10.1523/JNEUROSCI.2637-10.2010 21084593 PMC2997622

[B107] Riviere G. R. Riviere K. H. Smith K. S. (2002). Molecular and immunological evidence of oral Treponema in the human brain and their association with Alzheimer’s disease. Oral Microbiol. Immunol. 17, 113–118. 10.1046/j.0902-0055.2001.00100.x 11929559

[B108] Rodríguez-Rodríguez E. Sánchez-Juan P. Mateo I. Infante J. Sánchez-Quintana C. García-Gorostiaga I.. (2008). Interaction between CD14 and LXRβ genes modulates Alzheimer’s disease risk. J. Neurol. Sci. 264, 97–99. 10.1016/j.jns.2007.08.001 17900622

[B109] Rossol M. Heine H. Meusch U. Quandt D. Klein C. Sweet M. J.. (2011). LPS-induced cytokine production in human monocytes and macrophages. Crit. Rev. Immunol. 31, 379–446. 10.1615/critrevimmunol.v31.i5.20 22142165

[B110] Ruitenberg A. den Heijer T. Bakker S. L. van Swieten J. C. Koudstaal P. J. Hofman A.. (2005). Cerebral hypoperfusion and clinical onset of dementia: the Rotterdam Study. Ann. Neurol. 57, 789–794. 10.1002/ana.20493 15929050

[B111] Sampson T. R. Debelius J. W. Thron T. Janssen S. Shastri G. G. Ilhan Z. E.. (2016). Gut microbiota regulate motor deficits and neuroinflammation in a model of Parkinson’s disease. Cell 167, 1469.e12–1480.e12. 10.1016/j.cell.2016.11.018 27912057 PMC5718049

[B112] Savva G. M. Wharton S. B. Ince P. G. Forster G. Matthews F. E. Brayne C. (2009). Age, neuropathology, and dementia. N. Engl. J. Med. 360, 2302–2309. 10.1056/NEJMoa0806142 19474427

[B113] Scheltens P. Barkhof F. Leys D. Wolters E. C. Ravid R. Kamphorst W. (1995). Histopathologic correlates of white matter changes on MRI in Alzheimer’s disease and normal aging. Neurology 45, 883–888. 10.1212/WNL.45.5.883 7746401

[B114] Schmidt R. Schmidt H. Curb J. D. Masaki K. White L. R. Launer L. J. (2002). Early inflammation and dementia: a 25-year follow-up of the Honolulu-Asia Aging Study. Ann. Neurol. 52, 168–174. 10.1002/ana.10265 12210786

[B115] Schneider J. A. Arvanitakis Z. Bang W. Bennett D. A. (2007). Mixed brain pathologies account for most dementia cases in community-dwelling older persons. Neurology 69, 2197–2204. 10.1212/01.WNL.0000271090.28148.24 17568013

[B116] Scott A. J. Oyler B. L. Goodlett D. R. Ernst R. K. (2017). Lipid A structural modifications in extreme conditions and identification of unique modifying enzymes to define the Toll-like receptor 4 structure-activity relationship. Biochim. Biophys. Acta 1862, 1439–1450. 10.1016/j.bbalip.2017.01.004 28108356 PMC5513793

[B117] Selkoe D. J. (2011). Alzheimer’s disease. Cold Spring Harb. Perspect. Biol. 3:a004457. 10.1101/cshperspect.a004457 21576255 PMC3119915

[B118] Selmaj K. W. Raine C. S. (1988). Tumor necrosis factor mediates myelin and oligodendrocyte damage *in vitro*. Ann. Neurol. 23, 339–346. 10.1002/ana.410230405 3132891

[B119] Sharon G. Sampson T. R. Geschwind D. H. Mazmanian S. K. (2016). The central nervous system and the gut microbiome. Cell 167, 915–932. 10.1016/j.cell.2016.10.027 27814521 PMC5127403

[B120] Sheline Y. I. Morris J. C. Snyder A. Z. Price J. L. Yan Z. D’Angelo G.. (2010). APOE4 allele disrupts resting state fMRI connectivity in the absence of amyloid plaques or decreased CSF Aβ42. J. Neurosci. 30, 17035–17040. 10.1523/JNEUROSCI.3987-10.2010 21159973 PMC3023180

[B121] Shen L. Liu L. Ji H. F. (2017). Alzheimer’s disease histological and behavioral manifestations in transgenic mice correlate with specific gut microbiome state. J. Alzheimers Dis. 56, 385–390. 10.3233/JAD-160884 27911317

[B122] Sheng J. G. Bora S. H. Xu G. Borchelt D. R. Price D. L. Koliatsos V. E. (2003). Lipopolysaccharide-induced-neuroinflammation increases intracellular accumulation of amyloid precursor protein and amyloid β peptide in APPswe transgenic mice. Neurobiol. Dis. 14, 133–145. 10.1016/s0969-9961(03)00069-x 13678674

[B123] Stein P. S. Desrosiers M. Donegan S. J. Yepes J. F. Kryscio R. J. (2007). Tooth loss, dementia and neuropathology in the Nun study. J. Am. Dent. Assoc. 138, 1314–1322; quiz 1381–1312. 10.14219/jada.archive.2007.0046 17908844

[B124] Tan Z. S. Beiser A. S. Vasan R. S. Roubenoff R. Dinarello C. A. Harris T. B.. (2007). Inflammatory markers and the risk of Alzheimer disease: the Framingham Study. Neurology 68, 1902–1908. 10.1212/01.WNL.0000263217.36439.da 17536046

[B125] Tilvis R. S. Kähönen-Väre M. H. Jolkkonen J. Valvanne J. Pitkala K. H. Strandberg T. E. (2004). Predictors of cognitive decline and mortality of aged people over a 10-year period. J. Gerontol. A Biol. Sci. Med. Sci. 59, 268–274. 10.1093/gerona/59.3.m268 15031312

[B126] Tyas S. L. Manfreda J. Strain L. A. Montgomery P. R. (2001). Risk factors for Alzheimer’s disease: a population-based, longitudinal study in Manitoba, Canada. Int. J. Epidemiol. 30, 590–597. 10.1093/ije/30.3.590 11416089

[B127] Ujiie M. Dickstein D. L. Carlow D. A. Jefferies W. A. (2003). Blood-brain barrier permeability precedes senile plaque formation in an Alzheimer disease model. Microcirculation 10, 463–470. 10.1038/sj.mn.7800212 14745459

[B501] van de Haar H. J. Burgmans S. Jansen J. F. van Osch M. J. van Buchem M. A. Muller M.. (2016a). Blood-brain barrier leakage in patients with early Alzheimer disease. Radiology 281, 527–535. 10.1148/radiol.2016152244 27243267

[B502] van de Haar H. J. Jansen J. F. A. van Osch M. J. P. van Buchem M. A. Muller M. Wong S. M.. (2016b). Neurovascular unit impairment in early Alzheimer’s disease measured with magnetic resonance imaging. Neurobiol. Aging 45, 190–196. 10.1016/j.neurobiolaging.2016.06.006 27459939

[B503] van de Haar H. J. Jansen J. F. A. Jeukens C. R. L. P. N. Burgmans S. van Buchem M. A. Muller M.. (2017). Subtle blood-brain barrier leakage rate and spatial extent: considerations for dynamic contrast-enhanced MRI. Med. Phys. 44, 4112–4125. 10.1002/mp.12328 28493613

[B128] Veerhuis R. (2011). Histological and direct evidence for the role of complement in the neuroinflammation of AD. Curr. Alzheimer Res. 8, 34–58. 10.2174/156720511794604589 21143154

[B129] Verma S. Nakaoke R. Dohgu S. Banks W. A. (2006). Release of cytokines by brain endothelial cells: a polarized response to lipopolysaccharide. Brain Behav. Immun. 20, 449–455. 10.1016/j.bbi.2005.10.005 16309883

[B130] Verreault R. Laurin D. Lindsay J. De Serres G. (2001). Past exposure to vaccines and subsequent risk of Alzheimer’s disease. CMAJ 165, 1495–1498. 11762573 PMC81665

[B131] Veurink G. Fuller S. J. Atwood C. S. Martins R. N. (2003). Genetics, lifestyle and the roles of amyloid β and oxidative stress in Alzheimer’s disease. Ann. Hum. Biol. 30, 639–667. 10.1080/03014460310001620144 14675907

[B132] Villegas-Llerena C. Phillips A. Garcia-Reitboeck P. Hardy J. Pocock J. M. (2016). Microglial genes regulating neuroinflammation in the progression of Alzheimer’s disease. Curr. Opin. Neurobiol. 36, 74–81. 10.1016/j.conb.2015.10.004 26517285

[B133] Vlad S. C. Miller D. R. Kowall N. W. Felson D. T. (2008). Protective effects of NSAIDs on the development of Alzheimer disease. Neurology 70, 1672–1677. 10.1212/01.WNL.0000311269.57716.63 18458226 PMC2758242

[B134] Vogt N. M. Kerby R. L. Dill-McFarland K. A. Harding S. J. Merluzzi A. P. Johnson S. C.. (2017). Gut microbiome alterations in Alzheimer’s disease. Sci. Rep. 7:13537. 10.1038/s41598-017-13601-y 29051531 PMC5648830

[B135] Vollmar P. Kullmann J. S. Thilo B. Claussen M. C. Rothhammer V. Jacobi H.. (2010). Active immunization with amyloid-β 1–42 impairs memory performance through TLR2/4-dependent activation of the innate immune system. J. Immunol. 185, 6338–6347. 10.4049/jimmunol.1001765 20943998

[B136] Walter S. Letiembre M. Liu Y. Heine H. Penke B. Hao W.. (2007). Role of the toll-like receptor 4 in neuroinflammation in Alzheimer’s disease. Cell. Physiol. Biochem. 20, 947–956. 10.1159/000110455 17982277

[B137] White L. Small B. J. Petrovitch H. Ross G. W. Masaki K. Abbott R. D.. (2005). Recent clinical-pathologic research on the causes of dementia in late life: update from the Honolulu-Asia Aging Study. J. Geriatr. Psychiatry Neurol. 18, 224–227. 10.1177/0891988705281872 16306244

[B138] Winek K. Dirnagl U. Meisel A. (2016). The gut microbiome as therapeutic target in central nervous system diseases: implications for stroke. Neurotherapeutics 13, 762–774. 10.1007/s13311-016-0475-x 27714645 PMC5081128

[B140] Wu Z. Ni J. Liu Y. Teeling J. L. Takayama F. Collcutt A.. (2017). Cathepsin B plays a critical role in inducing Alzheimer’s disease-like phenotypes following chronic systemic exposure to lipopolysaccharide from Porphyromonas gingivalis in mice. Brain Behav. Immun. 65, 350–361. 10.1016/j.bbi.2017.06.002 28610747

[B141] Wu Z. C. Yu J. T. Li Y. Tan L. (2012). Clusterin in Alzheimer’s disease. Adv. Clin. Chem. 56, 155–173. 10.1016/B978-0-12-394317-0.00011-X 22397031

[B139] Wu D. Zhang X. Zhao M. Zhou A. L. (2015). The role of the TLR4/NF-κB signaling pathway in Aβ accumulation in primary hippocampal neurons. Sheng Li Xue Bao 67, 319–328. 26109305

[B142] Xie D. Shen F. He S. Chen M. Han Q. Fang M.. (2016). IL-1β induces hypomyelination in the periventricular white matter through inhibition of oligodendrocyte progenitor cell maturation via FYN/MEK/ERK signaling pathway in septic neonatal rats. Glia 64, 583–602. 10.1002/glia.22950 26678483

[B143] Zenaro E. Pietronigro E. Della Bianca V. Piacentino G. Marongiu L. Budui S.. (2015). Neutrophils promote Alzheimer’s disease-like pathology and cognitive decline via LFA-1 integrin. Nat. Med. 21, 880–886. 10.1038/nm.3913 26214837

[B144] Zhan X. Cox C. Ander B. P. Liu D. Stamova B. Jin L. W.. (2015a). Inflammation combined with ischemia produces myelin injury and plaque-like aggregates of myelin, amyloid-β and AβPP in adult rat brain. J. Alzheimers Dis. 46, 507–523. 10.3233/JAD-143072 25790832 PMC4878315

[B146] Zhan X. Jickling G. C. Ander B. P. Stamova B. Liu D. Kao P. F.. (2015b). Myelin basic protein associates with AβPP, Aβ1–42, and amyloid plaques in cortex of Alzheimer’s disease brain. J. Alzheimers Dis. 44, 1213–1229. 10.3233/JAD-142013 25697841 PMC4422390

[B145] Zhan X. Jickling G. C. Ander B. P. Liu D. Stamova B. Cox C.. (2014). Myelin injury and degraded myelin vesicles in Alzheimer’s disease. Curr. Alzheimer Res. 11, 232–238. 10.2174/1567205011666140131120922 24484278 PMC4066812

[B147] Zhan X. Stamova B. Jin L. W. DeCarli C. Phinney B. Sharp F. R. (2016). Gram-negative bacterial molecules associate with Alzheimer disease pathology. Neurology 87, 2324–2332. 10.1212/WNL.0000000000003391 27784770 PMC5135029

[B148] Zhang R. Miller R. G. Gascon R. Champion S. Katz J. Lancero M.. (2009). Circulating endotoxin and systemic immune activation in sporadic amyotrophic lateral sclerosis (sALS). J. Neuroimmunol. 206, 121–124. 10.1016/j.jneuroim.2008.09.017 19013651 PMC2995297

[B150] Zhao Y. Cong L. Jaber V. Lukiw W. J. (2017a). Microbiome-derived lipopolysaccharide enriched in the perinuclear region of Alzheimer’s disease brain. Front. Immunol. 8:1064. 10.3389/fimmu.2017.01064 28928740 PMC5591429

[B151] Zhao Y. Jaber V. Lukiw W. J. (2017b). Secretory products of the human gi tract microbiome and their potential impact on Alzheimer’s disease (AD): detection of lipopolysaccharide (LPS) in AD hippocampus. Front. Cell. Infect. Microbiol. 7:318. 10.3389/fcimb.2017.00318 28744452 PMC5504724

[B149] Zhao Y. Lukiw W. J. (2015). Microbiome-generated amyloid and potential impact on amyloidogenesis in Alzheimer’s disease (AD). J. Nat. Sci. 1:e138. 26097896 PMC4469284

[B152] Zhao Y. Zhao H. Lobo N. Guo X. Gentleman S. M. Ma D. (2014). Celastrol enhances cell viability and inhibits amyloid-β production induced by lipopolysaccharide *in vitro*. J. Alzheimers Dis. 41, 835–844. 10.3233/JAD-131799 24685625

[B153] Zhou H. Lapointe B. M. Clark S. R. Zbytnuik L. Kubes P. (2006). A requirement for microglial TLR4 in leukocyte recruitment into brain in response to lipopolysaccharide. J. Immunol. 177, 8103–8110. 10.4049/jimmunol.177.11.8103 17114485

[B154] Zhu X. Smith M. A. Honda K. Aliev G. Moreira P. I. Nunomura A.. (2007). Vascular oxidative stress in Alzheimer disease. J. Neurol. Sci. 257, 240–246. 10.1016/j.jns.2007.01.039 17337008 PMC1952687

[B155] Zlokovic B. V. (2005). Neurovascular mechanisms of Alzheimer’s neurodegeneration. Trends Neurosci. 28, 202–208. 10.1016/j.tins.2005.02.001 15808355

[B156] Zlokovic B. V. (2008). The blood-brain barrier in health and chronic neurodegenerative disorders. Neuron 57, 178–201. 10.1016/j.neuron.2008.01.003 18215617

[B157] Zlokovic B. V. (2011). Neurovascular pathways to neurodegeneration in Alzheimer’s disease and other disorders. Nat. Rev. Neurosci. 12, 723–738. 10.1038/nrn3114 22048062 PMC4036520

[B158] Zlokovic B. V. Martel C. L. Matsubara E. McComb J. G. Zheng G. McCluskey R. T.. (1996). Glycoprotein 330/megalin: probable role in receptor-mediated transport of apolipoprotein J alone and in a complex with Alzheimer disease amyloid β at the blood-brain and blood-cerebrospinal fluid barriers. Proc. Natl. Acad. Sci. U S A 93, 4229–4234. 10.1073/pnas.93.9.4229 8633046 PMC39517

